# Double cascade dressed MOSFET from doped Eu^3+^and Pr^3+^ in a host YPO_4_

**DOI:** 10.1039/c9ra08550e

**Published:** 2019-11-27

**Authors:** Huanrong Fan, Al Imran, Faizan Raza, Irfan ahmed, Kamran Amjad, Peng Li, Yanpeng Zhang

**Affiliations:** Key Laboratory for Physical Electronics and Devices of the Ministry of Education, Shaanxi Key Lab of Information Photonic Techniques, Xi'an Jiaotong University Xi'an 710049 China ypzhang@mail.xjtu.edu.cn; Department of Physics, City University of Hong Kong Hong Kong; Electrical Engineering Department, Sukkur IBA Sukkur 65200 Sindh Pakistan

## Abstract

In this paper, we study double cascade dressed optical metal oxide semiconductor field-effect transistor (MOSFET) by exploiting enhancement and suppression for mixed-phase (hexagonal + tetragonal) of Eu^3+^:YPO_4_ and different phases (hexagonal + tetragonal and pure tetragonal) of Pr^3+^:YPO_4_ crystals. We report variation of fine structure energy levels in different doped ions (Eu^3+^ and Pr^3+^) in the host YPO crystal. We compared multi-level energy transition from a single dressing laser with single level energy transition from double cascade dressing lasers. Gate delay facilitates multi-energy level dressed transition and is modeled through a Hamiltonian. Based on the results of double cascade dressing, we have realized MOSFET for logic gates (inverter and logic not and gate) with a switching contrast of about 92% using a mixed phase of Pr^3+^:YPO_4_.

## Abstract 2

By changing different parameters of single and double lasers, we observed the dressed energy level transition from single to multi-level with a single laser. Each sample responded based on their ions' structure and phase symmetry in the host YPO crystal.

## Graphical abstract

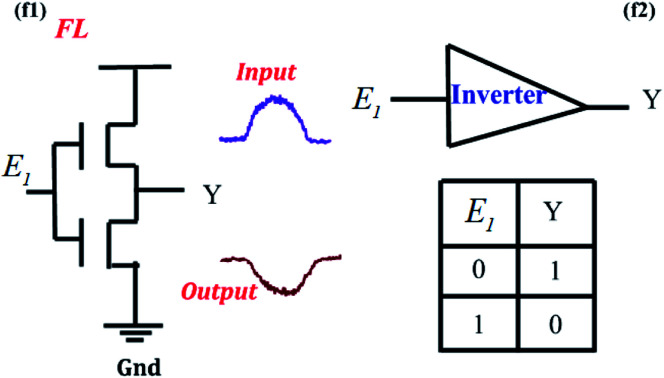

## Introduction

In the last few decades, scientists have shown increased interest in widening the knowledge of rare earth ions doped in crystals due to the potential applications in optical devices^1–5^ and quantum computing.^6^ Since Eu^3+^ and Pr^3+^ ions are more sensitive to the site symmetry and its surrounding crystal-field of the host material than other crystal ions,^7,8^ it can be achievable to get such kinds of potential application in YPO_4_ crystals. The crystal structure of YPO_4_ has two polymorphic forms, tetragonal (T-) and hexahedral (H-) phases.^9–12^ The slight variation of local structure will bring significant changes in the optical properties.

In theory, the tetragonal (T) phase of crystal is more structurally symmetric than the hexahedral (H) phase in Eu^3+^ and Pr^3+^ ions because of a more atomic-like system.^13^ In our experiment, a mixed contribution of T phase (more) + H phase (less) of crystal at low power performed better because of good transmission of information for the crystal T phase. The atomic density has a great influence on the number of splitting, which is relative to the dressing effect in atomic-like media.^14,15^ The observation of Autler–Townes (AT) splitting effect of FL spectrum induced by self or external fields and the polarization dependence of FL signals in Pr^3+^:Y_2_SiO_5_ has been reported.^16^ Wen *et al.*, realized optical switch and amplifier from dressing suppression and enhancement inmulti-order fluorescence (FL) and spontaneous parametric four-wave mixing (SP-FWM) in Pr^3+^:Y_2_SiO_5_.^17^ Controlled correlation and squeezing in Pr^3+^:Y_2_SiO_5_ to yield correlated light beams has also been investigated.^18^ Transition between the bright and dark states can modify the non-linear behaviour of crystal on singly- and doubly-dressed four-wave-mixing (FWM) processes.^19^ Diamond nitrogen-vacancy (NV) center were studied to realize optical transistors and hybrid switch.^20,21^ Eu^3+^:YPO_4_ and Pr^3+^:YPO_4_ crystals have been configured to observe second-order FL signals.

In this paper, we study the energy level transition of europium doped YPO (Eu^3+^:YPO_4_) and praseodymium doped YPO (Pr^3+^:YPO_4_) crystals with different phases using dressing lasers. By changing different parameters of single and double laser (power, detuning, and gate delay), we observed the dressed energy level transition from single to multi-level with a single laser; and single-level using double laser. Each sample responded based on their ions structure and phase symmetry in the host YPO crystal. In contrast to single multi-level dressing, double cascade dressed single level energy transition outputs are robust and are observed to be of special interest because of half peak with half suppression dip. These kinds of results are very important for realizing metal oxide semiconductor field-effect transistor (MOSFET).

## Basic theory

**Fig. 1:**
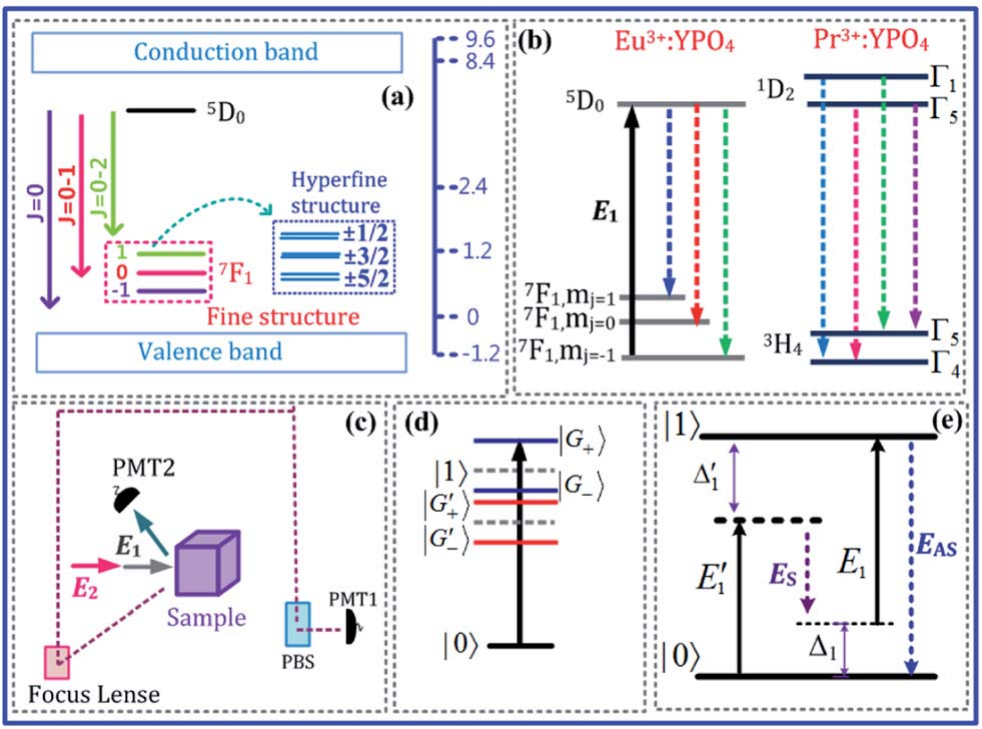
(a) Fine and hyperfine energy level of Eu^3+^:YPO_4_. (b) Energy level diagram of Eu^3+^:YPO_4_ and Pr^3+^:YPO_4_.^22–24^ (c) Schematic diagram of experimental setup. (d) Four-wave mixing (FWM) of lambda level system. (e) FWM in a two-level system.

In this experiment, both samples were held separately during the experiment running in a cryostat (CFM-102) with a temperature of 77 K. Fig. 1(a) shows a fine structure and a hyperfine structure of host material YPO_4_. The Eu^3+^:YPO_4_ has two states, named ground state ^7^F_1_ and excited state ^5^D_0_. Under the action of the crystal field of YPO_4_, the ground state ^7^F_1_ is split, which is fine-structure levels. Fig. 1(b) shows the simplified energy-level diagram of Eu^3+^ doped YPO_4_ (Eu^3+^:YPO_4_) and Pr^3+^ doped YPO_4_ (Pr^3+^:YPO_4_) crystal. Fig. 1(c) shows the schematic diagram of the experimental setup. Two dye lasers (narrow scan with a 0.04 cm^−1^ line width) are pumped by an injection-locked single-mode Nd:YAG laser (Continuum Powerlite DLS 9010, 10 Hz repetition rate, 5 ns pulse width), which are used to generate the pumping fields *E*_1_ (*ω*_1_, *Δ*_1_) and *E*_2_ (*ω*_2_, *Δ*_2_) with frequency detuning of *Δ*_*i*_ = *Ω*_mn_ − *ω*_*i*_, where *Ω*_mn_ is the corresponding atomic transition frequency between levels |m〉〉 and |n〉. *ω*_*i*_ (*i* = 1, 2) is the laser frequency. Arrangements of two photomultiplier tubes (PMT1-2) are used to detect the generated Stokes (*E*_S_), anti-Stokes (*E*_AS_)_,_ and FL composite signals (Fig. 1(c)). The pumping field *E*_*i*_ (where *i* = 1, 2) excites the sample and is reflected field 
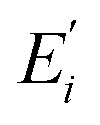
 from the surface of Eu^3+^:YPO_4_ into its original with a small angle *θ* between them. The spectral signals are obtained by scanning laser frequency, while time-domain signals are obtained by fixing laser frequency. Fig. 1(d) and (e) show dressed energy level, through which SP-FWM process configured in the lambda level system. Fig. 1(e) SP-FWM in a two-level system.

In Fig. 1(e), the density matrix of the FL *via* perturbation chain 
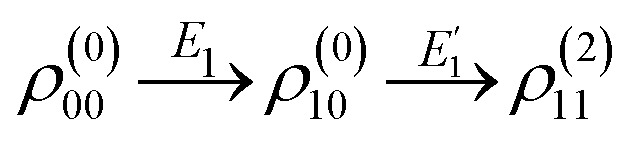
 can be written as

1 *ρ*^(2)^_11_ = −|*G*_1_|^2^/[(*d*_1_ + |*G*_1_|^2^/*Γ*_00_)(*Γ*_11_ + |*G*_1_|^2^/*d*_1_)],

where *d*_1_ = *Γ*_10_ + i*Δ*_1_, *G*_*i*_ = −*μ*_*ij*_*E*_*i*_/*ℏ* is the Rabi frequency, *Γ*_*ij*_is the transverse decay rate and *μ*_*ij*_ is the electric dipole moment between levels |*i〉* and |*j〉* the lifetime of FL is given as *Γ*_FL_ = *Γ*_10_ + *Γ*_11_. The temporal intensity of FL, given as *I*(*t*) = *ρ*^(2)^_11_exp(−*Γ*_FL_*t*). In a two-level system (shown in Fig. 1(e)), by opening field *E*_1_, the density matrix for the *E*_S_ and *E*_AS_ signals from hexagonal-phase of Eu^3+^:YPO_4_*via* perturbation chains 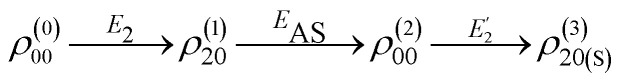 and 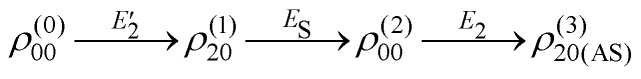, respectively, can be written as2
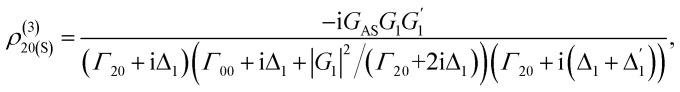
3
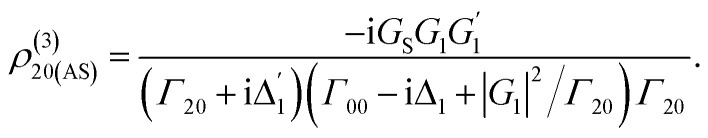


The lifetime of the Stokes/anti-Stokes signal can be written as *Γ*_S/AS_ = *Γ*_00_ + 2*Γ*_20_.

In Λ-type three-level system(shown in Fig. 4(c)), taking into account the self-dressing effect of *E*_1_ and the external-dressing field *E*_2_, the third-order nonlinear density matrix elements of *E*_S_ and *E*_AS_ are given by 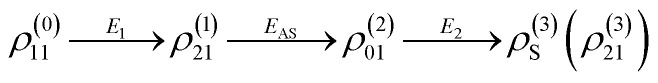 and 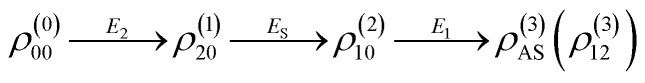, respectively. The dressed density matrix elements, in this case, are given as follows4
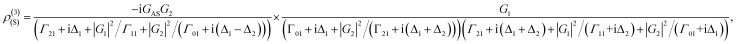
5
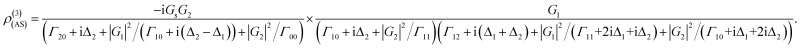


The lifetime of the Stokes/anti-Stokes signal can be written as *Γ*_AS_ = *Γ*_10_ + 2*Γ*_20_. Similarly, fourth-order FL *ρ*^(4)^_FL_ in Λ-type system *via* the pathway 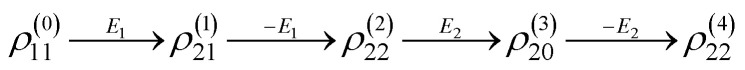 is

6 *ρ*^(4)^_FL_ = −|*G*_1_|^2^/[(*Γ*_21_ + i*Δ*_1_)(*Γ*_22_ + |*G*_1_|^2^/(*Γ*_01_ − i*Δ*_1_) + |*G*_2_|^2^/(*Γ*_20_ + i*Δ*_2_))].

Therefore, the intensity of the measured FL signal can be described as7
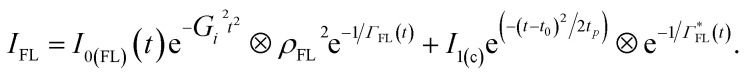


Owing to the interaction of the coupling field, the homogeneous linewidth broadening of the measured for FL is given as

8 *Γ*_*i*/*j*_ = *Γ*_pop_ + *Γ*_ion–spin_ + *Γ*_ion–ion_ + *Γ*_phonon_ − *Γ*_dressing_.

## Experimental results and discussion

**Fig. 2:**
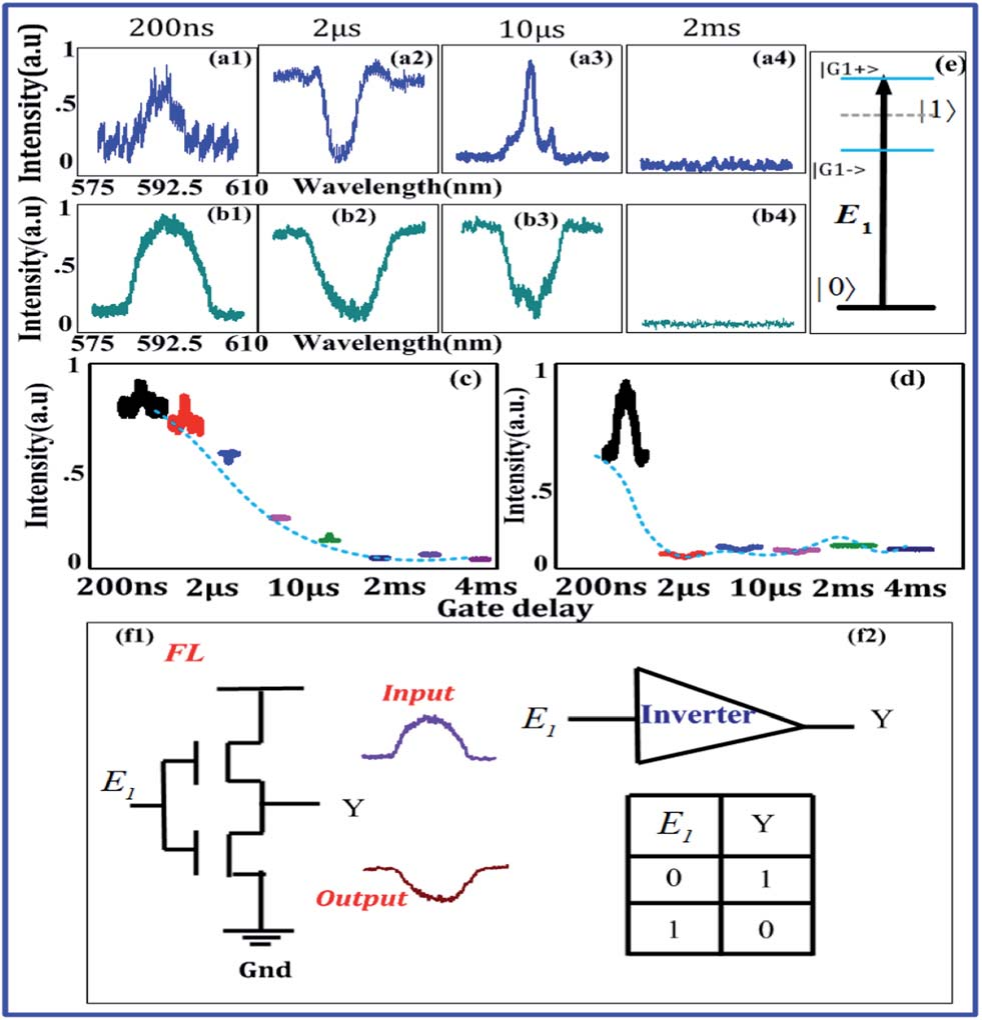
(a1–a4) Shows spectral intensity observed from a mixed phase of Eu^3+^ (more tetragonal and less hexagonal) doped in YPO at different gate delays and with fixed *E*_1_ at 4 mW while scanned from 575 nm to 610 nm. (b1–b4) Shows the same as (a1–a4), respectively, but the spectral signal, in this case, is routed through confocal lens to PMT (confocal detector). (c) and (d) Connecting diagram for PMT2 and confocal PMT1 corresponding to (a) and (b), respectively. (e) Energy level diagram for a single laser. (f) Schematic diagram of the MOSFET logic “not” gate.

Fig. 2(a1–a4) and (b1–b4) show spectral intensities of output signals observed at PMT2 and PMT1 (confocal detector), respectively, form a mixed phase of Eu^3+^:YPO_4_. The intensities displayed from Fig. 2(a1 and b1)–(a4 and b4) are recorded by gate delay at 200 ns, 2 μs, 10 μs, and 2 ms, respectively. Change in gate delay is demonstrated in Fig. 2(c) and (d); corresponds to change in different energy levels of Fig. 2(a) and (b). The bright to dark states are shown in Fig. 2(e) for illustration purpose based on single laser and single-level dressing. In Fig. 2(a1 and a3), the spectral signal recorded at PMT2 is composed of second-order FL (*ρ*^(2)^_11_) and SP-FWM (*ρ*^(3)^_10(S/AS)_), thus making a compo 
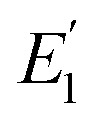
 site signal (*ρ*^(2)^_11_ + *ρ*^(3)^_S_) at PMT2. Composite signal is by produced *E*_1_ and 
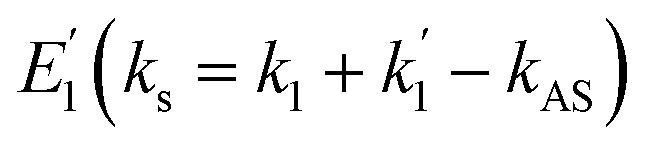
 along with FL at PMT2, and Fig. 2(b1) is similar to PMT1. In comparison with Fig. 2(a1), FL dominates in Fig. 2(b1) due to the focused detection. Physically, Hamiltonian is at |1〉 a frequency reference point (dark state) in Fig. 2(e). Using this equation of Hamiltonian *H*|*G*_1±_〉 = *λ*_±_|*G*_1±_〉, one can get the split energy level 
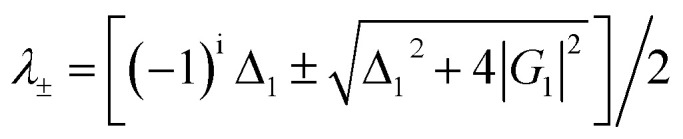
. When gate delay is changed from 200 ns to 2 μs, the dip appears in Fig. 2(a2) due to dressed suppression conditions *Δ*_1_ = 0of (|*G*_1_|^2^*/d*_3_) from eqn (2). When the gate delay is at 2 μs and 10 μs, the dip also appears in Fig. 2(b2 and b3). This phenomenon can be explained by *E*_1_ falling on dark states, which splits into two bright states (|*G*_1+_〉 and |*G*_1−_〉) and one dark state (|1〉), likewise the case in Fig. 2(a2). Signal linewidth is decreased from gate delay 200 ns to 10 μs. in Fig. 2(a1 and a3) due to SP-FWM *ρ*^(3)^_S_ in *ρ*^(2)^_11_ + *ρ*^(3)^_S_. At the gate delay of 2 ms, the intensity of the spectral signal is decreased. So, only the intensity noise signal is obtained in Fig. 2(a4). The single dressing effect of *E*_1_ is different at PMT1 or PMT2 by changing gate delay at medium power. Comparing recorded spectral intensities at PMT1 and PMT2, PMT1 has more FL due to detection through confocal lens, demonstrating broader line width and relatively obvious single laser dressing.

Here, an optical MOSFET based logic inverter or “not” gate has been realized through the results of Fig. 2(a) and (b). The model of the MOSFET logic inverter gate is shown in Fig. 2(f), where *E*_*1*_ is input signal (analogous to the gate voltage and gate current of MOSFET) and *Y* is the output of the MOSFET. To realize the logic inverter or not gate function of the MOSFET, when the input of the MOSFET *E*_1_ performs off-state, the output of the MOSFET *Y* performs on-state as a spectral peak in Fig. 2(a1) and (a3) (likewise Fig. 2(f1)). Here in Fig. 2(a1) and (a3), the output of the MOSFET *Y* satisfies the logical output condition 1. The spectral intensity of output spectral signal (off-states) in Fig. 2(a2) responses like MOSFET inverter as a suppression dip (Fig. 2(f2)). The MOSFET inverter as a suppression dip performs off state in Fig. 2(a2) (likewise Fig. 2(f2)), and it satisfies the output *Y* of the MOSFET inverter with logical 0 condition. The logic inverter or not gate contrast can be defined as *C* = (*I*_off_ − *I*_on_)/(*I*_off_ + *I*_on_), where *I*_off_ is the light intensity at the off-state and *I*_on_ is the light intensity at the on-state. The switching contrast *C* is about 75% from Fig. 2(a2)–(a3). Compared with Fig. 2(a2)–(a3), the logic inverter, or not gate contrast in Fig. 2(b1)–(b2) is similar, and the contrast is almost 78% from Fig. 2(b1)–(b2). The speed of the MOSFET inverter gate is 3 μs and 12 ns from Fig. 2(a1)–(a2) and from Fig. 2(b1)–(b2), respectively.

**Fig. 3:**
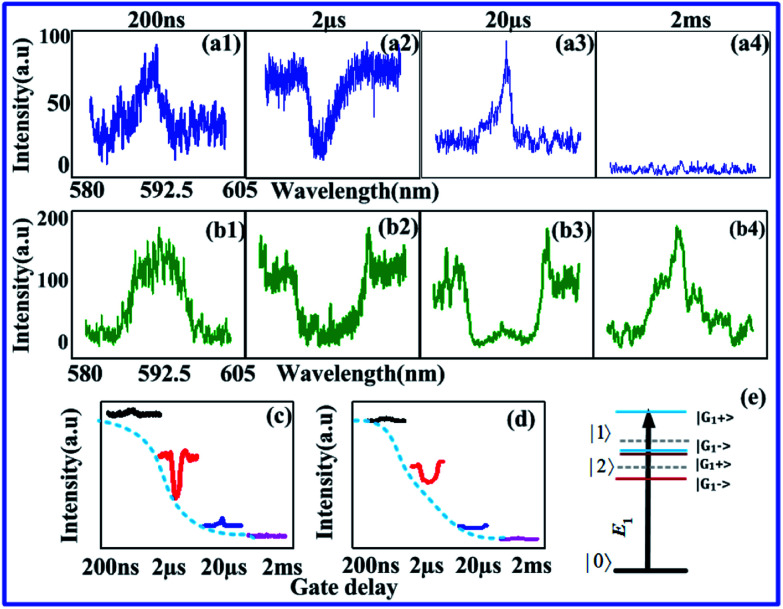
(a1–a4) and (b1–b4) Show spectral signal of Eu^3+^:YPO_4_ (more tetragonal and less hexagonal) from detector 2 at low 1 mW and high 8 mW respectively, with different gate delays of 200 ns, 2 μs, 20 μs and 5 ms at 77 K. (c and d) Shows the same as (c) and (d), respectively, but at power of laser field changed to 8 mW. (e) Shows the multi-energy level diagram for two single individual dressing from a single laser beam.

Fig. 3(a1–a4) and (b1–b4) shows spectral intensities of output signals measured at PMT2, when *E*_1_ is fixed at low power (1 mW) and high power (8 mW), respectively. Signal linewidth is also decreased from gate delays of 200 ns to 20 μs in Fig. 3(a1, a3) and (b1, b4) due to *ρ*^(3)^_S_. But signal linewidth in Fig. 3(b1–b4) at 8 mW is wider than that of in Fig. 3(a1–a4) at 1 mW due to multi-energy level dressed split at higher power by single dressing laser (Fig. 3(e)). However, at focused PMT1, the FL demonstrated the robust behaviour affected by dressing laser and gate delay dependency of multi-energy levels. It should be noted that the multi-level energy splitting is caused by *E*_1_ only, whereas, higher gate delay act as assisting to dressing parameter, which opens up a window for multi-energy level dressing, causing splitting as the two phases are closely packed in mixed-phase symmetry of Eu^3+^:YPO_4_. In principle, gate delay facilitates *E*_1_ field dressing, which excites (|1〉 and |2〉) and splits them into four bright states and two dark states, as illustrated in Fig. 3(e). In comparison with Fig. 2(e), two dark states |1〉 and |2〉 in Fig. 3(e) are different from one dark state |1〉. So one can say that |*G*_1±_〉 of |1〉 and |*G*_1±_〉 of |2〉form four bright states from a single dressing *E*_1_ when facilitated by gate delay. Physically, when the laser power of *E*_1_ is high, dressing |*G*_1_|^2^*/d*_1_ is increased suggested by eqn (1). By looking at Fig. 3(b3) at 20 μs, one can say laser dressing is assisted by gate delay to produce one small sharp peak, which comes by combining bright states (|*G*_1−_〉 of |1〉 and |*G*_1+_〉 of |2〉), and two dips (|1〉 and |2〉) from Fig. 3(e). One dip is shown in Fig. 3(a2 and b2), this phenomenon can be explained likewise Fig. 2(a2, b2 and b3). The interpretation of this peak in Fig. 3(a1, a3, b1 and b4) is same as in Fig. 2(a1, a3 and b1). Therefore, when the gate delay is increased at low power, the single dressing appears in Fig. 3(a3). At high power, dressing assisted by gate delay shows in Fig. 3(b2 and b3).

The MOSFET logic inverter or not gate performs on state, and it satisfies the output *Y* of the MOSFET inverter's logic 1 condition in Fig. 3(a1) and (b1). The output of the MOSFET *Y* performs off-state when it satisfies the logical output 0 condition in Fig. 3(a2) and (b2). So, MOSFET logic inverter or not gate is realized from Fig. 3(a1)–(a2) and from Fig. 3(b1)–(b2), where the inverter contrast *C* is about 72% and 80%, respectively.

**Fig. 4:**
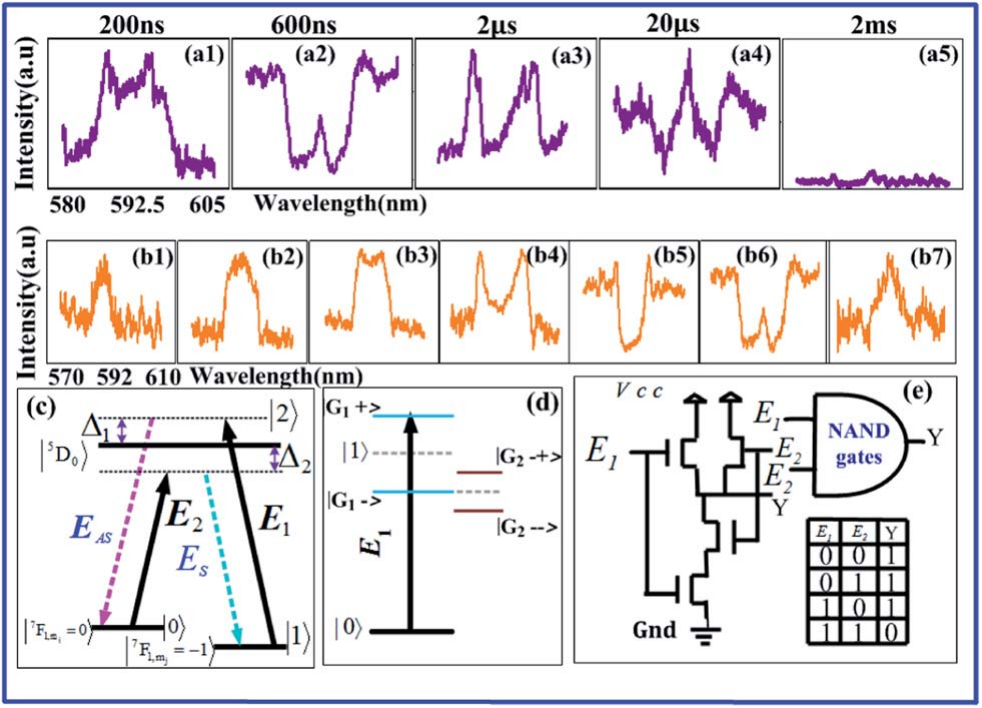
(a1–a5) Shows the spectral intensity recorded from a mixed phase of more tetragonal and less hexagonal Eu^3+^:YPO_4_ focused PMT1 by employing *E*_1_ and *E*_2_ at 77 K with gate delays of 200 ns, 600 ns, 2 μs, 20 μs, and 2 ms, respectively and at 8 mW of *E*_2_. (b1–b7) Shows the same recorded signal by changing *E*_1_ power from 2 mW to 8 mW while scanning *E*_2_ from 570–610 nm. (c) Lambda level system. (d) The energy level for double cascade dressing. (e) Schematic diagram of the NAND gate.

In Fig. 4(a1–a4), when gate delay is changed to 600 μs from 200 μs, the effect of double cascade dressing of *E*_1_ and *E*_2_ is getting obvious in Fig. 4(a2 and a4). The double dressing of *E*_1_ and *E*_2_ fall in dark states, which splits into three bright states (|*G*_1+_〉, |*G*_2−+_〉 and |*G*_2−−_〉) demonstrating three visible peaks and two dark state (|1〉, |*G*_1−_〉) corresponding to two dips in Fig. 4(a2 and a4), as suggested by Fig. 4(d). These dips and peaks are suggested by double cascade dressing |*G*_1_|^2^/(*Γ*_01_ − i*Δ*_1_) + |*G*_2_|^2^/(*Γ*_00_ + i*Δ*_2_) due to dressing suppression conditions *Δ*_1_ = 0, *Δ*_2_ = *G*_1_ from eqn (6). Fig. 4(a1 and a3) shows that by increasing gate delay from 200 ns to 2 μs AT splitting area varies from plan to deep. As dressing *E*_1_ plays a major role in Fig. 4(a1), while dressing *E*_1_ plays more role than dressing *E*_2_ in Fig. 4(a3). In Fig. 4(b1–b7), when *E*_1_ (594.8 nm) is shined by changing power from 2 mW to 8 mW and scanning *E*_2_ from 570–610 nm with 8 mW of power, similar trend of three visible peaks and two dips are gradually observed from Fig. 4(b6 and b7). AT splitting area varies from plan to deep by increasing power from 3 mW to 6 mW. The more obvious dressed energy level appears in Fig. 4(b2–b5) due to dressing *E*_2_ of high power. Comparing these two phenomenon of obtaining SP-FWM from suppressed dip of FL using gate delay in Fig. 4(a1–a5) and changing powers in Fig. 4(b1–b7), one can find the stronger double cascade dressing with more tetragonal and less hexagonal phase of Eu^3+^:YPO_4_ at 20 μs due to symmetry in Fig. 4(b6 and b7). Dressing effect of *E*_1_ and *E*_2_ in terms of AT splitting is obvious by changing gate delay and power in Eu^3+^:YPO_4_. Therefore one can characterize such a phenomenon obtained in Fig. 2(a2, b2 and b3), Fig. 3(b3) and Fig. 4(a2, b6 and b7) as single dressing, multi-energy level dressing by single laser assisted by gate delay and double cascade dressing in Eu^3+^:YPO_4_, respectively.

Here, we have realized the optical logic NAND gate. The model of optical logic NAND gate is shown in Fig. 4(e). When *E*_1_ power is 2 mW, the intensity input spectral signal satisfies the logical condition (0,0) of the NAND gate, so the output spectral signal performs as on state (logical output 1) in Fig. 4(b1). When *E*_1_ power is increasing gradually from 2 mW to 4 mW, output spectral signals perform as on state in Fig. 4(b2–b3) and here, the input condition of a logical NAND gate can be (0,1) and (1,0) for realization. When *E*_1_ power is 6 mW, the intensity output spectral signal satisfies the logical input condition (1,1) of the NAND gate, and the output spectral signal performs as an off state (logical output 0) in Fig. 4(b5). Our experiment results are defined on state and off state by the NAND gate contrast, respectively inverter contrast. Here, *C* is 86% from Fig. 4(b1–b3) to (b5).

**Fig. 5:**
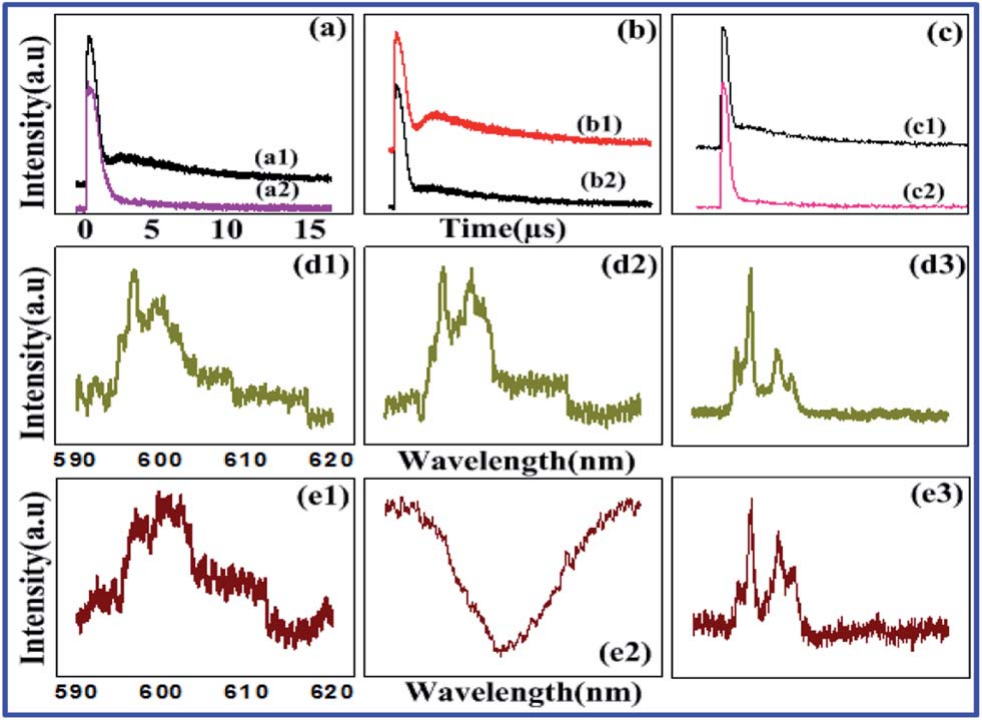
(a) Shows the temporal intensity obtained from pure tetragonal phase of Pr^3+^:YPO_4_ at 600 nm, recorded from PMT2 by keeping (a1) *E*_1_, *E*_2_ and (a2) *E*_2_ laser beam at 1 mW at 77 K. (b1 and b2) Shows the same as (a1 and a2), respectively, but at resonant (596.8 nm) excitation. (c) Same signal obtained through (c1) resonant excitation of *E*_1_ and (c2) Off-resonance (590 nm) excitation of *E*_1_. (d1–d3) shows spectral intensity from Pr^3+^:YPO_4_ at different gate position 500 ns, 1.5 μs and 5 μs, with single *E*_1_ and (e1–e3) both *E*_1_ and *E*_2_.

In Fig. 5(a1), the time-domain signal has little adiabatic population than Fig. 5(a2), because of double cascade dressing from *E*_1_ and *E*_2_. At resonant excitation in Fig. 5(b1 and b2), one can say that the adiabatic population is maximum at a resonant point in Fig. 5(b1) and comparatively less in Fig. 5(b2) because of a single dressing of *E*_2_. By looking at Fig. 5(c1 and c2), less adiabatic expansion has found for resonant and off-resonant *E*_1_ at 77 K. Signal linewidth is also decreased from the gate position 500 ns to 5 μs in Fig. 5(d1 and d3) or (e1 and e3) due to stokes of SP-FWM *ρ*^(3)^_S_. In the crystal field of YPO_4_:Pr^3+^, the (2*J* + 1) degeneracy is partly split, and the *J* = 0, 1, 2, 3, 4, 5, 6 levels split into 1, 2, 4, 5, 7, 8, 10 irreducible representations, respectively. Under this symmetry (^1^D_2_ and ^3^H_4_), two transition levels are allowed.^19^Fig. 5(e2) shows double cascade dressing at low power due to dressing suppression conditions *Δ*_1_ = 0, *Δ*_2_ = 0 from eqn (6). Double cascade dressing of *E*_1_ and *E*_2_ is more obvious than that of *E*_1_ at low power or even by changing gate delay in Pr^3+^:YPO_4_. By comparing Pr^3+^ and Eu^3+^, at low power, the double cascade dressing effect in Pr^3+^:YPO_4_ (in Fig. 5(e2)) is stronger than Eu^3+^:YPO_4_ (in Fig. 4(b2)).

Here in Fig. 5, optical logic NAND gate has been realized, respectively Fig. 4. In Fig. 5(e1), spectral signal performs output on state (logical output 1) from the input logical (0,0) condition of optical logic NAND gate, as shown in the model in Fig. 4(e). In Fig. 5(e2), spectral signal performs output off state (logical output 1), and it can satisfy the input logical (1,1) condition of optical logic NAND gate, as shown in the model in Fig. 4(e). Here, contrast *C* is 88% (from Fig. 5(e1)–(e2)) as gate delay changed from 1 μs to 1.5 μs.

**Fig. 6:**
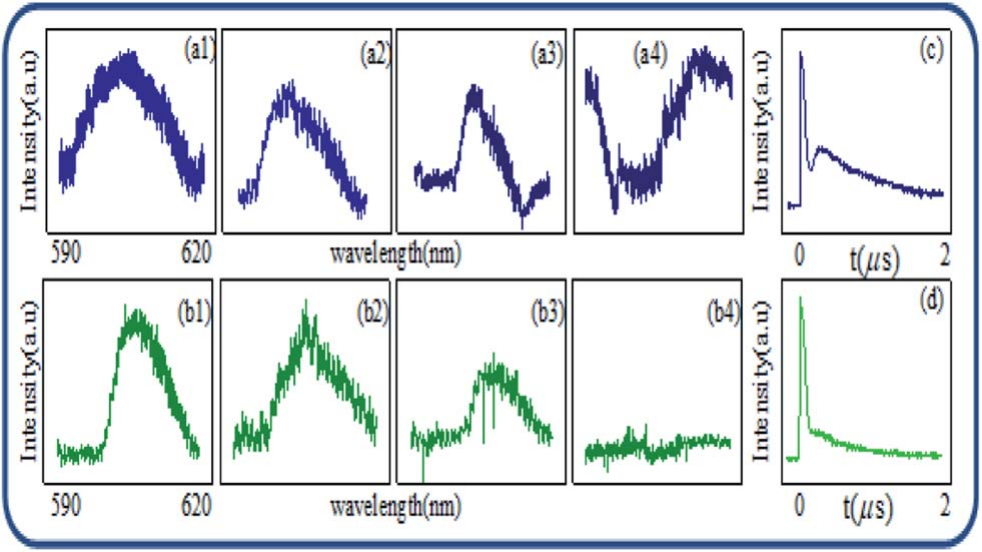
(a1–a4) Shows the spectral intensity observed from mixed phase of Pr^3+^ (less tetragonal and more hexahedral) doped in YPO_4_ crystal at 77 K, recorded at PMT2 by changing gate delay from 200 ns, 600 ns, 1 μs and to 1.5 μs, respectively, and keeping *E*_1_ and *E*_2_ at low power 1 mW. (b1–b4) Shows the spectral intensity of the observed signal at PMT1 by changing gate delay and keeping the power of *E*_1_ at 1 mW. (c) and (d) Shows the time domain signal corresponding to (a) and (b), respectively.

Fig. 6(a1–a4) shows the gradual and bit weak trend of the FL signal. By comparing Fig. 5 and 6 at low power, one can say that dressing effects observed in the tetragonal phase and mixed-phase (less tetragonal with more hexahedral) of Pr^3+^:YPO_4_ are different, which can be explained from different site symmetry. Here, we have realized the optical logic NAND gate. When output spectral signals are obtained at gate delays 200 ns, 600 ns, and 1 μs at low power of *E*_1_ and *E*_2_ in Fig. 6(a1–a3), spectral signals are performed as output on state (output 1) of the optical logical NAND gate. For realizing the output logical NAND gate, input conditions can be (0,0), (0,1) and (1,0) respectively Fig. 6(a1–a3). When output spectral signal is at gate delay 1.5 μs at low power of *E*_1_ and *E*_2_ in Fig. 6(a4), NAND gate performs off state (output 0) and it can satisfy the input condition of logical (1,1) for the NAND gate. Our experiment result defined the NAND gate contrast as *C* is 92% as gate delay changed from 200 ns to 1.5 μs in Fig. 6(a1) and (a4).

## Conclusions

In summary, we demonstrated and compared single dressing based multi-level energy dressing assisted by gate delay and double cascade dressing based single energy-level transition in Eu^3+^:YPO_4_ and Pr^3+^:YPO_4_ crystals. We observed that at low power, a single dressing effect in Pr^3+^:YPO_4_ was stronger than Eu^3+^:YPO_4_, while the double cascade dressing effect was stronger in Eu^3+^:YPO_4_. Based on the outputs, logic gates (inverter and logic not and (NAND) gate) were realized by gate delay and power of laser fields. Such a detailed comparison of Eu^3+^ and Pr^3+^ doped in YPO_4_ can be of potential utility in nonlinear and quantum optics for quantum gates.

## Conflicts of interest

There are no conflicts to declare.

## Supplementary Material
